# First identification of bovine hepacivirus in wild boars

**DOI:** 10.1038/s41598-022-15928-7

**Published:** 2022-07-08

**Authors:** Claudio de Martinis, Lorena Cardillo, Claudia Esposito, Maurizio Viscardi, Lorella Barca, Stefania Cavallo, Nicola D’Alessio, Vito Martella, Giovanna Fusco

**Affiliations:** 1 Unit of Exotic and Vector-Borne Diseases, Department of Animal Health, Istituto Zooprofilattico Sperimentale del Mezzogiorno, Via Salute, 2, 80055 Portici, Naples, Italy; 2 Istituto Zooprofilattico Sperimentale del Mezzogiorno, Calabria Section, Cosenza, Italy; 3 Department of Epidemiologic and Biostatistic Regional Observatory (OREB), Istituto Zooprofilattico Sperimentale del Mezzogiorno, Portici, Naples, Italy; 4 Department of Veterinary Medicine, Aldo Moro” University, Bari, Italy

**Keywords:** Ecology, Microbiology, Molecular biology

## Abstract

Hepatitis C virus (HCV) is a major cause of chronic hepatitis, cirrhosis and hepatocellular carcinoma in humans. Humans were long considered the only hosts of *Hepacivirus*. Recently HCV-like sequences have been found in several animal species. Hepaciviruses are considered species-specific but a wider host range and a zoonotic role has been hypothesized. We report the first detection of bovine hepacivirus (BovHepV) sequences in wild boars. A total of 310 wild boars hunted in Campania region were investigated with a pan-hepacivirus nested-PCR protocol for the NS3 gene. Hepacivirus RNA was detected in 5.8% of the animals. Sequence and phylogenetic analysis showed high homology with BovHepV subtype F, with nucleotide identity of 99%. The positive wild boars were georeferenced, revealing high density of livestock farms, with no clear distinction between animal husbandry and hunting areas. These findings might suggest the ability of BovHepV to cross the host-species barrier and infect wild boars.

## Introduction

Hepatitis C virus (HCV) is described as one of the main cause of chronic hepatitis, cirrhosis and hepatocellular carcinoma^1^, with viral hepatitis-related mortality accounted for 48%^2^. Indeed, World Health Organization (WHO), in the last global hepatitis report, estimated HCV to affect 71 million people with chronic hepatitis with approximately 399 thousand deaths, mostly from cirrhosis and hepatocellular carcinoma^3^. HCV is an enveloped, linear positive single-stranded RNA virus, belonging to the *Flaviviridae* family, genus *Hepacivirus* that is characterized by hepatotropism and lymphotropism^4^. Humans were first considered the only hosts of *Hepacivirus* but, in the last decade, HCV-like sequences have been found in several animal species, such as dogs, bats, horses, rodents and sea animals^5–10^. Moreover, in 2015, a new *Hepacivirus* was described in cattle in Ghana and Germany^11,12^ that was classified as Bovine Hepacivirus (BovHepV). These new HCV-like viruses have been assigned to new *Hepacivirus* species, named from A to N, that show distinct host ranges^13,14^ and have maintained their hepatotropism^14^ and, more recently, two novel and divergent species of the genus Hepacivirus have been identified in squirrels and ducks, provisionally named as *Hepacivirus P* and *Q*, respectively^15,16^.

BovHepV is the only member of *Hepacivirus N* species^4,17^ and is supposed to infect cattle only^18^ although virus infection has not been associated with overt clinical signs^14^. It has been detected in bovine serum samples in several countries with a prevalence of infection ranging from 0.15% to 14.8% and in commercial bovine sera used for cell cultures in Asia, America, Oceania and Europe, thus suggesting a worldwide circulation^14,17^. BovHepV is characterized by high genetic heterogeneity, resulting in several clusters/subtypes named from A to G^14,17^, moreover, other recent strains detected in blood-sucking ticks of cattle, have been identified as a novel subtype H^19^. Nevertheless, the knowledge about the host range, the zoonotic potential and host tropism of this virus are still unclear^20^.

In this study we report the detection of BovHepV in wild boars. Wildlife and, in the specific, wild boar, can be considered as either a sentinel animal^21^ or as a possible vector^22^, for a number of emerging and re-emerging pathologies of zoonotic interest, chiefly in territories where a clear division between woodland-rural and urban environment is no longer present due to high population and housing density. Much remains to be investigated regarding the possible involvement of wild boar in the role of reservoir for pathogens with zoonotic potential.

## Results

During the hunting season 2019–2020, 310 wild boars were hunted in the five provinces of Campania region, in particular, 117 (37.7%) in Salerno, 85 (27.4%) in Benevento, 75 (24.2%) in Avellino, 29 (9.3%) in Caserta and 4 (1.3%) in Napoli province.

Each animal was submitted to *post mortem* inspection by veterinarian inspectors that sampled a total of 562 organs, 285 (50.7%) livers and 277(49.3%) muscles. Pan-hepacivirus RNA was detected in 20 (6.1%) organs from 19 animals (5.8%). Eighteen animals tested positive either in the muscular tissues (n = 11 or in the liver (n = 7), whilst 1 animal was positive in both the liver and muscle tissues. The province distribution of positive animals is summarized in Table 1.

**Table 1:** Province distribution of pan-hepacivirus in wild boars.

Province	Wild boar	Positive	%
Salerno	117	8	6.8
Benevento	85	2	2.3
Avellino	75	8	10.6
Caserta	29	1	3.4
Napoli	4	–	–
Total	310	19	6.1

**Figure 1:**
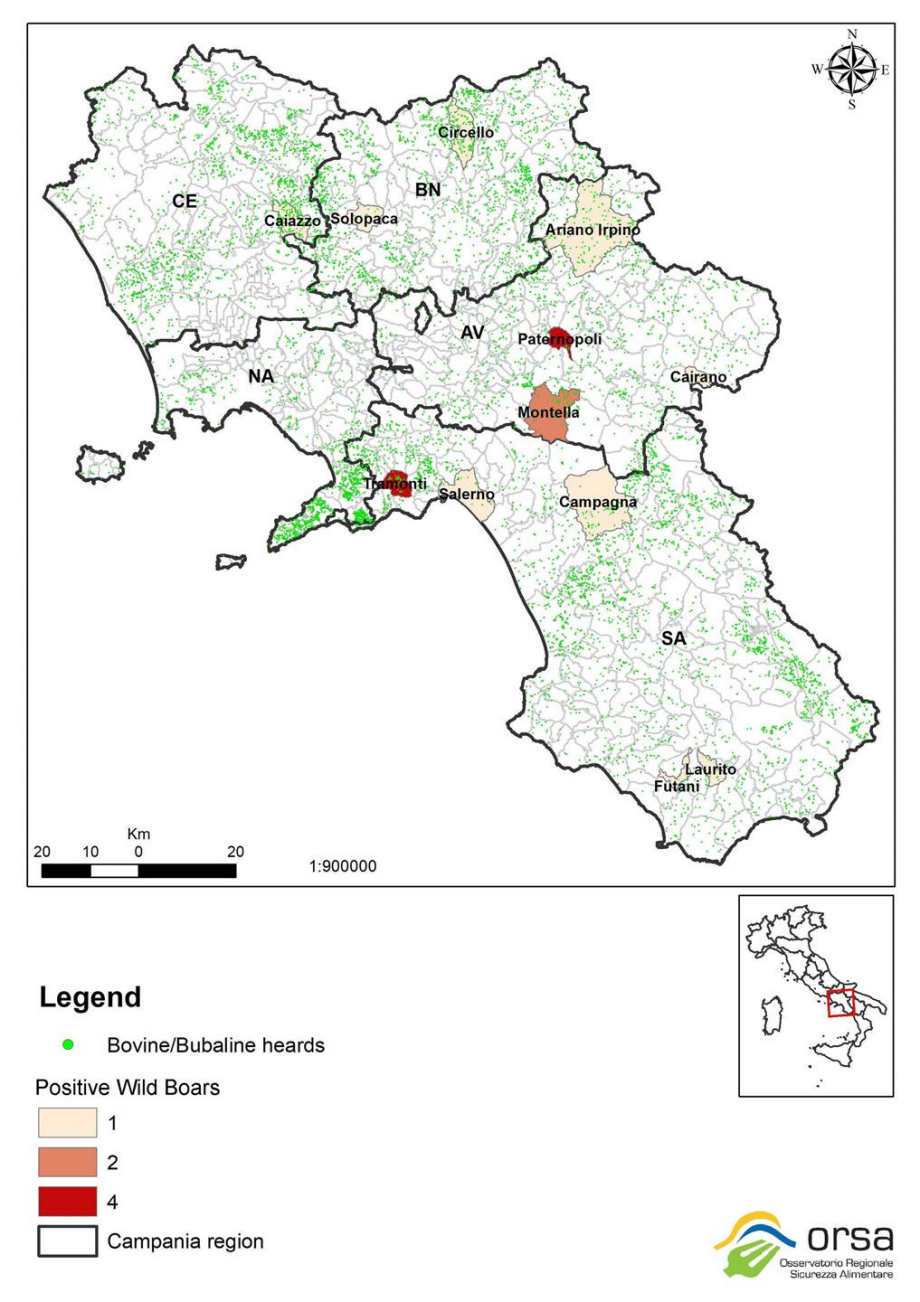
Distribution of tested positive wild boars and bovine-bubaline herds.

In order to evaluate the distribution of cattle and buffalo livestock farms throughout the Campania region and municipalities where the animals were hunted together with the number of tested positive wild boars, a georeferenced map was obtained (Fig. 1).

Figure 1 The georeferenced map was obtained by preparing a Geographical Information System (GIS), highlighting municipalities, tested positive wild boars and the distribution of bovine/bubaline herds by using ArcMap software version 10.7, with epsg projection 32633—wgs 84/utm zone 33n spatial reference system.

It can be observed high density of livestock farms and a very high territorial density, with no clear distinction between areas dedicated to breeding, animal husbandry and areas intended for hunting.

Among the 20 positive samples, 7 partial NS3 sequences were successfully obtained from 3 muscle (Accession no. MW538518, MW538519 and MW538522) and 4 liver samples (Accession no. MW538520, MW538521, MW538523, MW538524).

**Figure 2:**
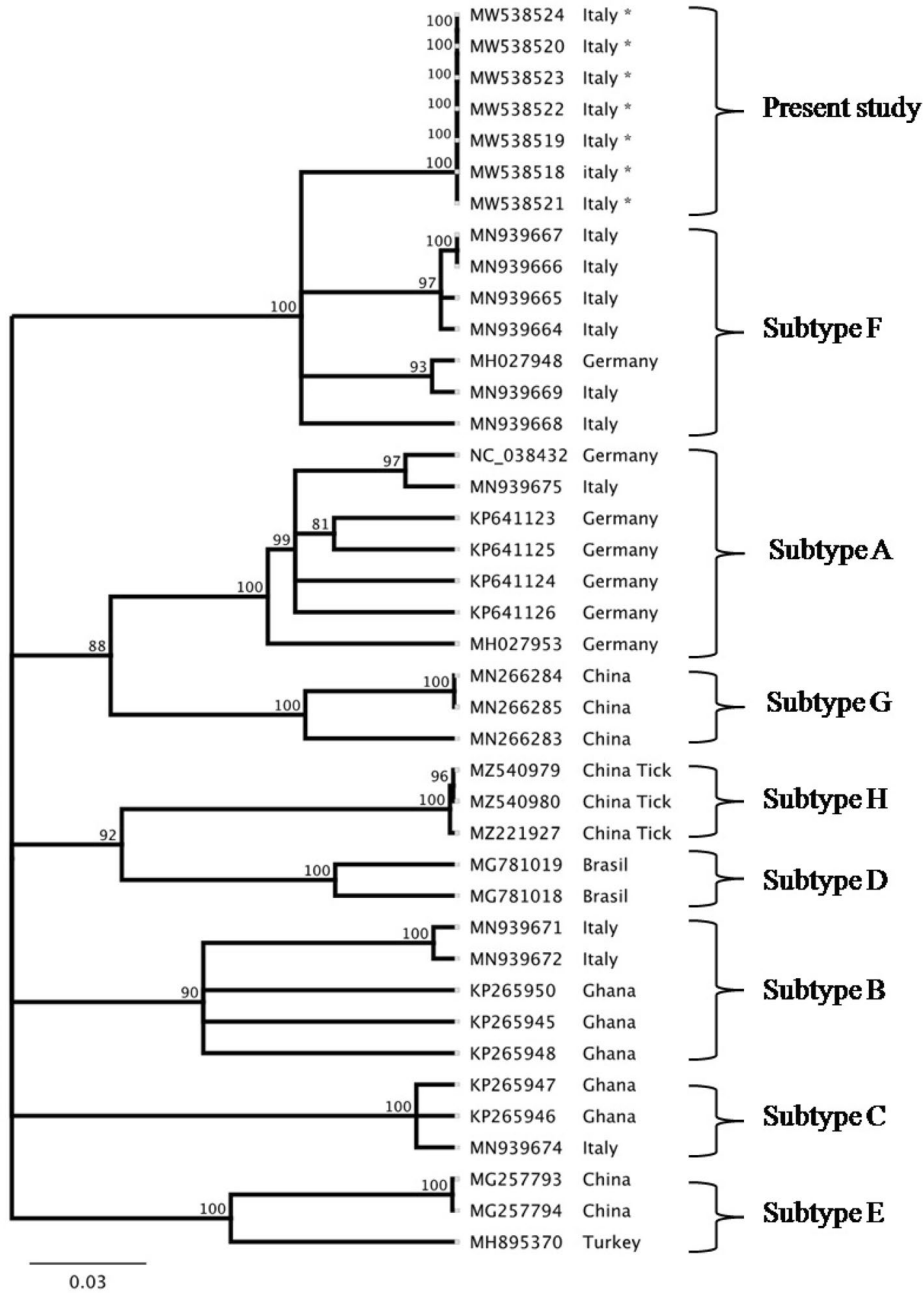
Phylogenetic analysis performed on BovHepV species NS3 gene.

Upon interrogation of GenBank sequence database, and evaluation of best nucleotide matches, different patterns of recognition were displayed by the 7 wild boar-associated BovHepV strains. The 7 sequences formed a distinct cluster, highly homogeneous genetically (0.0000 to 0.09432). This group of sequences segregated within the subtype F (0.16543), with other BovHepV strains found in Italy (MN939667, MN939666, MN939664, MN939665, MN939668, MN939669) and in Germany (MH027948) (Fig. 2).

Overall, the wild boar hepacivirus sequences seemingly formed a well-defined cluster within subtype F.

## Discussion

Hepatitis C virus has been long considered to be the unique species of *Hepacivirus* genus, restricted to the human host. However, since the early 2010s, new HCV-like have been described in several animal species and non-vertebrate hosts^5,15,16,19,23–25^. BovHepV was first described in 2015 by two independent research groups in Ghana and Germany^11,12^. Subsequently, several studies have demonstrated the presence of BovHepV in serum samples and in commercial sera for cell culture worldwide^14,17,26–28^. Some authors have supposed a possible wider host range and a potential zoonotic role for BovHepV, although this hypothesis has not been demonstrated, remaining at a speculative level^14,18,29^.

In the present study, evidence was obtained for the presence of BovHepV-like in wild boars, hinting to the possibility that these viruses are capable to infect other animal species. Interestingly, Baechlein and colleagues observed a serological reactivity in 2.5% swine sera using BovHepV NS3 luciferase immunoprecipitation system (LIPS), albeit no hepacivirus RNA was detected^20^.

Despite BovHepV is genetically heterogeneous^20^, on sequence analysis our findings showed the wild boar HCV-like sequences formed a single cluster, with high nucleotide identity to each other (100%) and to other strains belonging to subtype F (99%), already identified in Italy. This data could suggest a repeated spillover of a virus circulating in the area or a recent event of interspecies transmission between cattle and wild boar. The ability of HCV-like viruses to cross the species barrier is not a new element, as demonstrated by the genetic similarity between dog and horse hepaciviruses^30^. Furthermore, a large-scale study on hepacivirus evolution conducted by Thézé and colleagues, comparing the genome of HCV-like viruses identified in several animal species, revealed high genetic homology (only one different nucleotide) between canine and equine hepaciviruses from North America^31^. This could also be applied to our findings, showing high identity of HCV-like sequences detected in wild boars. A limit of our investigation is the unsuccessful attempt to generate whole genomic sequencing data from pooled liver and muscle samples with sequence-independent strategies that did not generate HCV-like reads, although this was not unexpected, considering the limits of sensitivity of metagenomic strategies. Moreover, considering the uncultivatable nature of HCV-like viruses^32^, cell-adaptation of the virus could not be pursued.

Wild boars are often proved to be biological indicators of other emerging/re-emerging diseases of zoonotic interest as well as a possible pathogen vector, either directly or as intermediate host^21,33,34^. Furthermore, high quality habitat in the landscape may promote either the contact between susceptible hosts and the infectious source, in the case of direct transmission, or environmental contamination, in the case of indirect transmission, creating the conditions for interspecific contagions^34^. The distribution of bovine and bubaline farms and the tested positive wild boars of our study, showed very high territorial density of livestock farms, mostly with extensive management, and overlapping breeding, animal husbandry and hunting areas. These conditions, especially in the presence of numerous extensive farms close to the wild, as for our case, could promote the promiscuity of domestic animals and wildlife. Indeed, wild boar behavior is characterized by constant search for food by moving up to 84 km per week^34^, also sharing fields and pastures with cattle. Thus, indirect contact with food, feces and wild animal carcasses, could easily lead intra / inter-species pathogen transmission^21,22^. In addition, the recently described possible involvement of blood-sucking ticks as mechanical carriers of BovHepV^19^, could also represent a further route of infection in both cattle and wild boars.

In conclusion, HCV virus is a major human pathogen. To date it is not clear whether BovHepV and HCV-like viruses could represent a zoonotic risk. However, the findings of our study extend the host range of HCV-like viruses. Attempts should be done to gather more sequence and epidemiological information on these viruses in wildlife.

## Methods

### Sample collection

The wild boar carcasses were transferred to specific hunting stations for the *post mortem* inspection performed by veterinary inspectors. Above all, a total of 285 livers and 277 muscles were obtained, stored in suitably closed containers and transferred as soon as possible to the Istituto Zooprofilattico Sperimentale del Mezzogiorno of Portici (IZSM), Naples, Italy. The organs were refrigerated at + 4 °C/+ 6 °C for 24–72 h or frozen. Tissue samples were obtained after cauterization of the surface, and excision of organs using sterile scalpels. Approximately 2 cm^3^ samples were collected from the inner parts of the organs and conducted to IZSM Virology labs.

### Nucleic acid extraction

For nucleic acid extraction, 2 mg of tissue samples were collected and suspended in 2 mL of sterile phosphate-buffered saline (PBS) in 2-mL tubes. Samples were homogenised with glass beads using a TissueLyser (Qiagen) and centrifuged for 5 min at 1700×*g*. Aliquots of 200 μL of the supernatant were collected and submitted to extraction and purification using QIAsymphony DSP Virus/Pathogen Mini Kit (Qiagen), performed by QIAsymphony automated system (Qiagen) following manufacturer’s instructions, eluted in 60 µL and stored at − 80 °C until use.

### Molecular detection and characterization of Bovine Hepacivirus

A nested pan-hepacivirus PCR was used according to the protocol of Baechlein et al.^20^, with minor modifications. Two sets of primers were used to amplify the highly conserved NS3 coding region. Primary PCR reaction was performed using PyroMark OneStep RT-PCR kit (Qiagen), giving rise to a 655 bp amplicon and, next, a nested PCR was performed using PyroMark PCR kit (Qiagen), that gave rise to a 328 bp amplicon. PCR amplifications were performed in a Mastercycler Nexus X2 thermal cycler (Eppendorf). Finally, 1 µL of the amplification product was analyzed by using D1000 screen tape and reagents (Agilent Technologies) for the capillary electrophoresis (Tapestation 2200, Agilent Technologies). Tested positive samples, were submitted to purification by using the MiniElute Reaction Cleanup kit (Qiagen), followed by Sanger sequencing, carried out by Big Dye Terminator Cycle Sequencing Kit v.1.1 (Applied Biosystems). Next, DyeEx 2.0 Spin kit (Qiagen) was used for the amplicon clean-up, and the sequencing reactions were applied to a 3500 Genetic Analyzer capillary electrophoresis system (Applied Biosystems). The forward and reverse sequences were assembled using the Geneious R9 software package (Biomatter) and compared to analogous sequences in the BLAST genetic database (http://www.ncbi.nlm.nih.gov/Blast.cgi). To exclude any possible contamination, a bovine serum positive to BovHepV, used as control, was sequenced, showing 92% nucleotide identity to the wild boar HCV-like sequences.

### Phylogenetic analysis

We calculated the best nucleotide substitution model for the dataset generated with the NS3 gene sequences obtained from GenBank. The evolutionary history was inferred by using the Maximum Likelihood method and Tamura-Nei model^35^. The tree with highest log likelihood (− 2072.84) is shown. The percentage of trees in which the associated taxa clustered together is shown next to the branches. Initial tree(s) for the heuristic search were obtained by applying the Neighbor-Joining method to a matrix of pairwise distances estimated by using the Maximum Composite Likelihood (MCL) approach. A discrete Gamma distribution was used to model evolutionary rate differences among sites (4 categories (+G, parameter = 0.7387)). The rate variation model allowed for some sites to be evolutionarily invariable ([+/], 18.99% sites). This analysis involved 38 nucleotide sequences. Codon positions included were 1st + 2nd + 3rd + Noncoding. There were a total of 244 positions in the final dataset. Evolutionary analyses were conducted in MEGA X^36^.

### Dataset

A georeferenced map was obtained by preparing a Geographical Information System (GIS), highlighting municipalities, tested positive wild boars and the distribution of bovine/bubaline herds. Data were carried out by the Epidemiologic and Biostatistic Regional Observatory (ORSA) by using ArcMap 10.7 software with epsg projection 32,633—wgs 84/utm zone 33n spatial reference system.

### Ethic statement

All investigators confirm that the ethical policies of the journal, as noted on the journal’s author guideline page have been adhered to. The authors ensure that the planning conduct and reporting of animal research are in accordance with the Helsinki Declaration as revised in 2013. Furthermore, the Istituto Zooprofilattico Sperimentale is the official lab designed by the Italian Ministry of Health. According to national regulation and international policy, ethical approval was deemed unnecessary since the sampling has been conducted by the local Competent Authorities during routine activities dictated by national and regional regulation.

## Data Availability

The datasets generated and analyzed during the current study are available in GenBank repository (https://www.ncbi.nlm.nih.gov/genbank/), Accession no. MW538518, MW538519, MW538522, MW538520, MW538521, MW538523, MW538524 [https://www.ncbi.nlm.nih.gov/nuccore/?term=bovine+hepacivirus+sus+scrofa].
